# Association between the serum albumin–creatinine ratio and 28-day intensive care unit mortality among patients with sepsis: a multicenter retrospective cohort study

**DOI:** 10.3389/fmed.2024.1484370

**Published:** 2024-11-05

**Authors:** Weiguo Lin, Cheng Fu, Jiangwei Miao, WeiLi Hong, Xinglin Chen, Shaorong Yan, Yuzhan Lin

**Affiliations:** ^1^Department of Urology, The Third Affiliated Hospital of Wenzhou Medical University, Zhejiang, China; ^2^Department of Clinical Laboratory, Ruian Traditional Chinese Medicine Hospital, Zhejiang, China; ^3^Department of Emergency Intensive Care Unit, The Third Affiliated Hospital of Wenzhou Medical University, Zhejiang, China; ^4^Department of Epidemiology and Biostatistics, Empower U, X&Y Solutions Inc., Boston, MA, United States; ^5^Department of Clinical Laboratory, The Third Affiliated Hospital of Wenzhou Medical University, Zhejiang, China

**Keywords:** sepsis, serum albumin–creatinine ratio, sACR, 28-day mortality, intensive care unit, ICU, retrospective cohort study

## Abstract

**Introduction:**

Sepsis is a substantial global health challenge with a considerable disease burden. Despite advancements in sepsis research, the mortality rates associated with this condition remain high. The relationship between the serum albumin-to-creatinine ratio (sACR) and mortality in patients with sepsis remains unclear. Therefore, this study aimed to investigate the association between the sACR and 28-day mortality in intensive care unit (ICU) patients with sepsis.

**Methods:**

In this retrospective cohort study, we used data sourced from the eICU Collaborative Research Database. The primary exposure variable was sACR, and the primary outcome measure was mortality within 28 days after ICU admission. Statistical analyses included univariate and multivariate logistic regression models, generalized additive models, and two-piecewise linear regression models, which were employed to explore non-linear relationships and threshold effects between sACR and mortality.

**Results:**

The study cohort comprised 9,690 ICU patients with sepsis, with a 28-day mortality rate of 9.99%. The results of the multivariate logistic regression model indicated that elevated sACR levels were significantly associated with a reduced risk of mortality (odds ratio = 0.78, 95% confidence interval: 0.71–0.87, *p* < 0.001), even after adjusting for potential confounding variables. Curve fitting revealed a non-linear relationship between sACR and 28-day mortality, with an inflection point of 4.79.

**Discussion:**

This study demonstrated that sACR is an independent risk factor for 28-day mortality in ICU patients with sepsis, exhibiting a non-linear negative dose–response relationship and a threshold effect. These findings may serve as early warning indicators in high-risk populations.

## 1 Introduction

Sepsis is a life-threatening organ dysfunction caused by a dysregulated host response to infection (1), and its global incidence is increasing. It is estimated that more than 30 million cases of sepsis occur worldwide each year, accounting for approximately one-quarter of all in-hospital deaths (2–4). Despite medical advancements, the hospital mortality rate for sepsis remains unacceptably high at 20%, escalating to more than 40% in cases of septic shock (2, 3, 5). These figures underscore the substantial threat that sepsis poses to global public health and highlight the urgent need for more effective treatments and in-depth research.

Serum albumin levels are closely associated with sepsis severity and prognosis (6, 7). Studies have demonstrated that hypoalbuminemia is associated with illness severity and an increased risk of mortality in patients with sepsis (8). Furthermore, the prevalence of acute kidney injury is high among patients with sepsis, and creatinine, a crucial indicator for assessing kidney function, plays a pivotal role in diagnosing sepsis-associated acute kidney injury (9). A multicenter observational study indicated that elevated creatinine levels were closely related to poor prognosis in patients with sepsis (10). In particular, kidney function affects serum albumin levels (11).

In light of the intricate interrelationships among sepsis, hypoalbuminemia, and kidney dysfunction, the incorporation of serum albumin and creatinine into sACR may offer a more comprehensive evaluation of alterations in sepsis. However, studies on sACR are limited. The limited findings that are currently available suggest that sACR less than the threshold of 3.75 is independently associated with an increased 1-year all-cause mortality risk. However, it should be noted that the study population was limited to intensive care unit (ICU) patients with heart failure (11). In addition, in a study of patients with acute myocardial infarction (AMI), Liu et al. found that sACR at admission was an independent predictor of all-cause and cardiac mortality, further emphasizing the value of sACR in the prognostic assessment of cardiac diseases (12). However, the relationship between sACR and mortality in ICU patients with sepsis remains unclear and requires further investigation. To address this research gap, we conducted a study using the U.S. eICU Collaborative Research Database (eICU-CRD), with the aim of exploring the association between sACR and 28-day mortality in ICU patients with sepsis.

## 2 Methods

### 2.1 Data source and ethics statements

In this retrospective cohort study, the data were sourced from eICU-CRD, an online international database in the United States (13). The data were accessed and extracted in compliance with the data usage protocol of the PhysioNet review committee, following examination and certification (record ID: 40859994). The database, jointly established by the eICU Institute and Massachusetts Institute of Technology’s Laboratory for Computational Physiology, is a vast public data resource encompassing more than 200,000 ICU patient records from 208 medical institutions in the United States between 2014 and 2015.

To ensure the protection of patient information and eliminate the need for additional informed consent from patients, all patient information involved in the study was de-identified. This study was conducted in accordance with the principles of the Declaration of Helsinki (14).

### 2.2 Study population

The study population comprised all patients hospitalized for sepsis. Sepsis was diagnosed based on the presence of a suspected or confirmed infection in conjunction with a rapid increase of more than 2 points in the Sequential Organ Failure Assessment (SOFA) score, derived from the Acute Physiology and Chronic Health Evaluation (APACHE) IV scale (4, 15). Infection identification was based on the International Classification of Diseases, Ninth Edition (ICD-9) codes recorded in the eICU-CRD. The following exclusion criteria were applied during the patient selection process: (1) patients not entering the ICU for the first time, (2) patients with ICU stays of less than 24 h, (3) patients younger than 18 years of age, (4) patients lacking ICU treatment outcome information, and (5) patients lacking sACR data or system recording errors.

### 2.3 Main study variables

The eICU database encompasses a wide array of patient information, including basic demographic data; physiological monitoring parameters from bedside monitors; diseases diagnosed according to the ICD-9, Clinical Modification (ICD-9-CM) codes; and other laboratory results collected during routine medical care. The research team concentrated their efforts on gathering data from the initial 24-h period following the patient’s admission. Vital sign data, including body temperature, respiratory rate, heart rate, and mean arterial pressure (MAP), were extracted from the Apache Aps Var table. Baseline patient characteristics, including age, sex, race, and body mass index (BMI), were collected from the patient and patient results tables. Comorbidities, including diabetes, liver failure, cirrhosis, and metastatic cancer, were identified using the APACHE IV scoring system. Relevant information was extracted from diagnostic tables. Laboratory indicators, including serum albumin, serum creatinine, alanine aminotransferase (ALT), aspartate aminotransferase (AST), and total cholesterol, were extracted from the laboratory table using SQL codes. We used the time of measurement (lab result offset) to select the first recorded values within 24 h of ICU admission as baseline indicators for analysis. The severity of the patient’s condition at admission was assessed using a range of scoring systems, including the SOFA, APACHE IV, Glasgow Coma Scale (GCS), and Acute Physiology Score III.

### 2.4 Outcome

The study outcome was the mortality rate within 28 days of ICU admission.

### 2.5 Statistical analysis

The patients were divided into four distinct cohorts, with each cohort corresponding to a quartile of sACR. Subsequently, a comprehensive description of the characteristics of each cohort was provided. Continuous variables that exhibited a normal distribution are expressed as mean ± standard deviation. By contrast, continuous variables that did not adhere to a normal distribution are expressed as median with interquartile range. One-way analysis of variance was used to compare normally distributed continuous variables across groups, whereas the non-parametric Kruskal–Wallis test was used for continuous variables that were not normally distributed. Numerical representations of categorical data are presented as count (*N*) and relative frequency (percentage). Statistical analyses of group disparities were conducted using the chi-square test.

Univariate and multivariate logistic regression models were used to estimate the correlation between sACR and 28-day mortality. The results are expressed as odds ratio (OR) with a 95% confidence interval (95% CI). The principle of adjusting covariates is determined based on clinical significance and change in the matched OR by at least 10% (16). The following covariates were adjusted for age, sex, BMI, ethnicity, temperature, respiratory rate, heart rate, MAP, blood urea nitrogen level, calcium level, AST level, ALT level, platelet count, hemoglobin level, white blood cell count, GCS score, SOFA score, chronic obstructive pulmonary disease, congestive heart failure, acute myocardial infarction, diabetes, pneumonia, hepatic failure, metastatic cancer, intubation status, mechanical ventilation use, and dialysis.

A generalized additive model (GAM) was used to investigate the dose–response relationship between sACR and 28-day mortality. Subsequently, a two-piecewise linear regression model was employed to investigate the threshold effect of sACR on mortality. When a clear relationship between sACR and 28-day mortality was observed on the smooth curve, software automatically calculated the inflection point using a recursive method after adjusting for potential confounders. Furthermore, a log-likelihood ratio test was conducted to compare univariate and two-piece linear regression models. Considering the heterogeneity of the population, we employed stratified linear regression models for subgroup analyses across different baseline indicators and presented the results as part of our sensitivity analysis. Statistical analyses were conducted using R software (version 4.2.0; R Foundation) and EmpowerStats software^1^ (X&Y Solutions, Inc., Boston, MA), and the level of statistical significance was set at a *p*-value of <0.05.

## 3 Results

### 3.1 Participants’ characteristics

**Figure 1:**
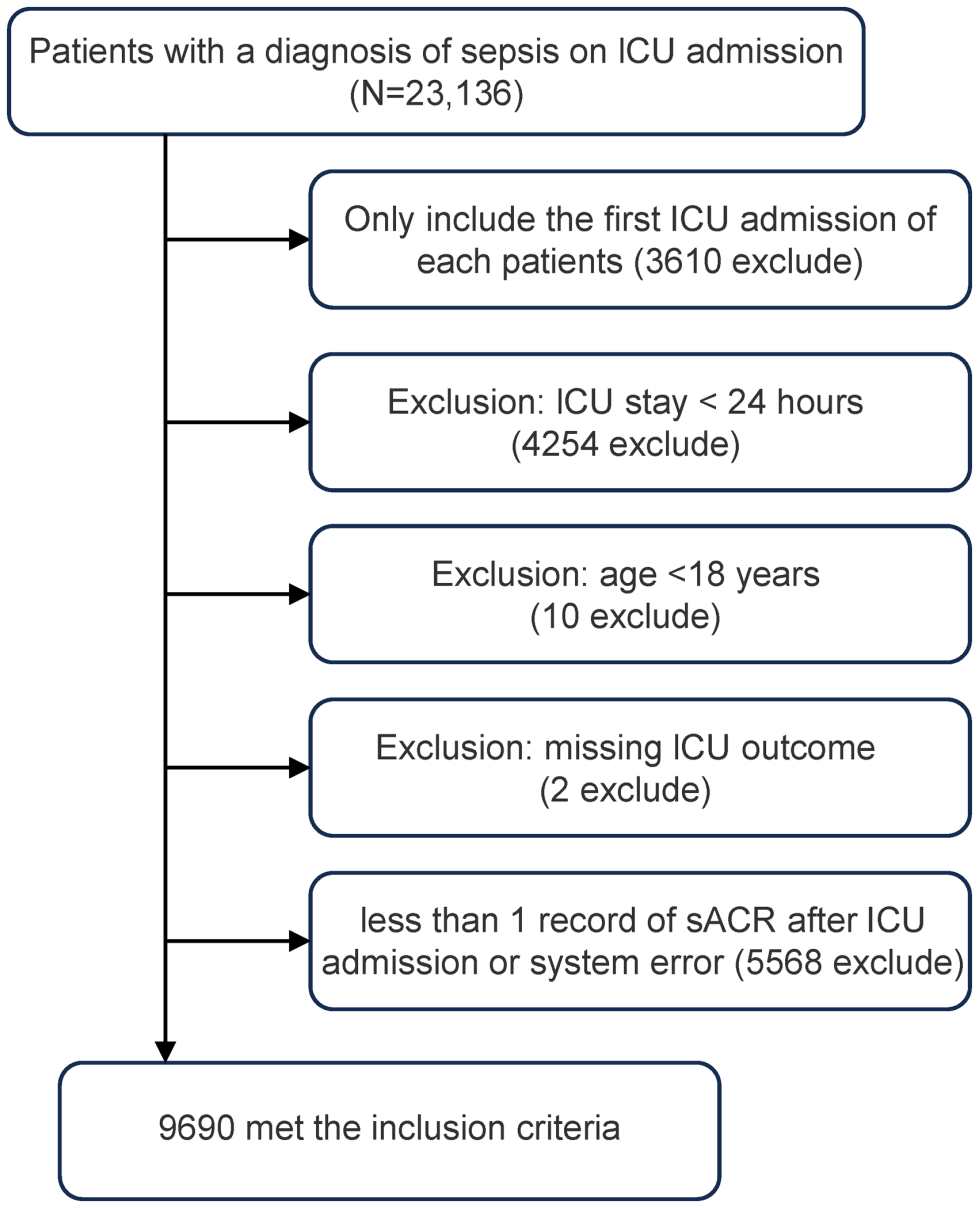
Flow chart of the subject inclusion.

A flowchart illustrating the study’s inclusion and exclusion criteria is shown in Figure 1. Table 1 presents a comprehensive account of the baseline characteristics of the 9,690 participants, including demographic data, vital signs, laboratory results, infection sites, comorbidities, and disease severity. The participants were divided into quartiles based on their sACR levels: Q1 (0.08–0.96), Q2 (0.96–1.74), Q3 (1.74–2.84), and Q4 (2.84–7.71). The group with lower sACR values exhibited a higher proportion of female patients (Q1: 1,355, 55.97%; Q2: 1,277, 52.73%; Q3: 1,306, 53.94%; Q4: 1,036, 42.72%) and patients with higher BMI (29.98 ± 9.26 kg/m^2^), along with significant increases in indicators of liver and kidney function impairment, including serum creatinine (Q1: 3.83, 2.93–5.41), ALT (Q1: 29.00, 17.00–70.00), and AST levels (Q1: 45.00, 23.00–119.25). Furthermore, these patients demonstrated markedly elevated Acute Physiology Score III, SOFA score, and APACHE IV score. The 28-day ICU mortality rates differed significantly among the groups (*p* < 0.001), with the number of deaths in each group as follows: Q1, 391 (16.15%); Q2, 283 (11.68%); Q3, 190 (7.85%); and Q4, 104 (4.29%).

**Table 1:** Baseline Characteristics of participants (*N* =9690).

Characteristic	sACR (quartiles)				
	**Q1(0.08-0.96)**	**Q2(0.96-1.74)**	**Q3(1.74-2.84)**	**Q4(2.84-7.71)**	*p*-value
No. of participants	2421	2423	2421	2425	
Demographics					
Age (years)	65.51 ± 14.69	68.76 ± 14.67	67.24 ± 15.52	61.25 ± 17.23	<0.001
Gender (N, %)					<0.001
Male	1066 (44.03)	1145 (47.27)	1115 (46.06)	1389 (57.28)	
Female	1355 (55.97)	1277 (52.73)	1306 (53.94)	1036 (42.72)	
Ethnicity (N, %)					<0.001
Caucasian	1728 (71.73)	1920 (79.77)	1947 (81.06)	1890 (78.59)	
African American	353 (14.65)	226 (9.39)	193 (8.03)	178 (7.40)	
Hispanic	135 (5.60)	106 (4.40)	122 (5.08)	162 (6.74)	
Asian	108 (4.48)	93 (3.86)	80 (3.33)	86 (3.58)	
Native American	36 (1.49)	15 (0.62)	22 (0.92)	31 (1.29)	
Other/Unknown	49 (2.03)	47 (1.95)	38 (1.58)	58 (2.41)	
BMI (kg/m^2^)	29.98 ± 9.26	29.60 ± 9.34	28.58 ± 8.44	27.54 ± 8.16	<0.001
Vital signs					
Temperature (°C)	36.35 ± 1.31	36.52 ± 1.35	36.67 ± 1.29	36.68 ± 1.12	<0.001
Respiratory rate (bpm)	29.07 ± 14.34	30.64 ± 14.21	30.74 ± 14.15	31.11 ± 14.53	<0.001
Heart rate (/min)	110.83 ± 29.08	111.27 ± 30.10	113.79 ± 28.72	114.09 ± 27.57	<0.001
MAP (mmHg)	74.63 ± 45.73	76.14 ± 44.72	75.24 ± 40.52	80.67 ± 40.89	<0.001
Laboratory data					
Lactate (mmol/l)	2.10 (1.30-3.70)	2.20 (1.40-3.60)	1.80 (1.20-2.80)	1.50 (1.00-2.30)	<0.001*
Triglycerides (mg/dl)	155.00 (109.00-219.00)	124.00 (86.00-192.00)	114.00 (75.00-170.00)	99.50 (65.75-144.00)	<0.001*
Total cholesterol (mg/dl)	98.50 (80.25-121.75)	102.00 (85.00-136.00)	103.50 (81.00-137.00)	112.00 (91.00-151.00)	0.009*
Albumin (g/dl)	2.28 ± 0.62	2.42 ± 0.57	2.52 ± 0.56	2.75 ± 0.57	<0.001
Serum creatinine (mg/dl)	3.83 (2.93-5.41)	1.90 (1.54-2.30)	1.17 (0.90-1.40)	0.69 (0.54-0.82)	<0.001*
Sodium (mmol/l)	137.16 ± 6.93	138.45 ± 6.59	138.80 ± 5.93	138.20 ± 5.75	<0.001
Calcium (mg/dl)	7.80 ± 1.00	7.87 ± 0.89	7.94 ± 0.80	8.07 ± 0.78	<0.001
Troponin I (ng/ml)	0.18 (0.07-0.81)	0.16 (0.06-0.69)	0.14 (0.04-0.71)	0.10 (0.04-0.39)	<0.001*
BNP (pg/ml)	1010.60 (372.00-3730.50)	828.00 (388.00-2148.50)	559.00 (215.00-1917.00)	332.00 (144.00-1055.00)	<0.001*
Blood urea nitrogen (mg/dl)	54.00 (39.00-76.00)	37.00 (27.00-50.00)	24.00 (17.00-33.00)	15.00 (10.00-21.00)	<0.001*
PT – INR	1.60 (1.30-2.30)	1.53 (1.30-2.20)	1.40 (1.20-2.00)	1.30 (1.10-1.70)	<0.001*
Platelets (x 10^9^/l)	168.00 (106.00-246.00)	170.00 (111.00-248.00)	176.00 (120.00-243.00)	199.00 (142.00-264.00)	<0.001*
Hemoglobin (g/dl)	9.92 ± 2.08	10.24 ± 2.15	10.52 ± 2.11	10.80 ± 2.16	<0.001
White blood cell count (×10^9^/l)	14.50 (9.30-21.60)	14.07 (9.00-20.54)	12.80 (8.50-18.40)	12.20 (8.20-17.50)	<0.001*
ESR (mm/hr)	55.50 (30.00-89.25)	53.00 (19.00-83.50)	51.50 (27.50-89.75)	34.00 (17.00-63.50)	0.017*
CRP (mg/dl)	21.10 (10.40-31.10)	23.35 (13.54-280.70)	18.30 (9.60-30.25)	17.16 (8.93-263.40)	0.149*
ALT, U/L	29.00 (17.00-70.00)	30.00 (17.00-64.00)	27.00 (17.00-50.00)	25.00 (15.50-46.00)	<0.001*
AST, U/L	45.00 (23.00-119.25)	42.00 (23.00-99.00)	34.00 (21.00-65.00)	29.00 (18.00-53.00)	<0.001*
Severity of illness					
Acute Physiology Score III	68.00 (55.00-88.00)	62.00 (49.00-78.00)	51.00 (40.00-66.00)	44.00 (34.00-59.00)	<0.001*
SOFA score	7.00 (5.00-9.00)	5.00 (3.00-7.00)	4.00 (2.00-6.00)	3.00 (1.00-4.00)	<0.001*
Apache IV score	82.00 (68.00-102.00)	78.00 (64.00-94.00)	66.00 (53.00-83.00)	57.00 (44.00-72.00)	<0.001*
GCS score	12.11 ± 3.67	12.34 ± 3.51	12.54 ± 3.42	12.60 ± 3.34	<0.001
Sepsis source of infection (N, %)					<0.001
Pulmonary	670 (27.67)	818 (33.76)	1021 (42.17)	1133 (46.72)	
Renal/UTI (including bladder)	582 (24.04)	650 (26.83)	501 (20.69)	448 (18.47)	
Gastrointestinal	408 (16.85)	346 (14.28)	337 (13.92)	283 (11.67)	
Cutaneous/Soft Tissue	230 (9.50)	196 (8.09)	160 (6.61)	172 (7.09)	
Gynecologic	5 (0.21)	6 (0.25)	6 (0.25)	12 (0.49)	
Unknown/Other	526 (21.73)	407 (16.80)	396 (16.36)	377 (15.55)	
Comorbidities					
Hepatic failure (N, %)					0.001
No	2321 (96.71)	2331 (96.76)	2337 (97.70)	2349 (98.24)	
Yes	79 (3.29)	78 (3.24)	55 (2.30)	42 (1.76)	
Metastatic cancer (N, %)					0.137
No	2332 (97.17)	2311 (95.93)	2308 (96.49)	2305 (96.40)	
Yes	68 (2.83)	98 (4.07)	84 (3.51)	86 (3.60)	
Cirrhosis (N, %)					<0.001
No	2289 (95.38)	2304 (95.64)	2315 (96.78)	2337 (97.74)	
Yes	111 (4.62)	105 (4.36)	77 (3.22)	54 (2.26)	
Diabetes (N, %)					<0.001
No	1602 (66.75)	1746 (72.48)	1820 (76.09)	1947 (81.43)	
Yes	798 (33.25)	663 (27.52)	572 (23.91)	444 (18.57)	
COPD (N, %)					<0.001
No	2284 (94.34)	2233 (92.16)	2217 (91.57)	2211 (91.18)	
Yes	137 (5.66)	190 (7.84)	204 (8.43)	214 (8.82)	
CHF (N, %)					<0.001
No	2227 (91.99)	2167 (89.43)	2211 (91.33)	2286 (94.27)	
Yes	194 (8.01)	256 (10.57)	210 (8.67)	139 (5.73)	
AMI (N, %)					0.070
No	2328 (96.16)	2332 (96.24)	2347 (96.94)	2360 (97.32)	
Yes	93 (3.84)	91 (3.76)	74 (3.06)	65 (2.68)	
Pneumonia (N, %)					<0.001
No	1813 (74.89)	1699 (70.12)	1539 (63.57)	1489 (61.40)	
Yes	608 (25.11)	724 (29.88)	882 (36.43)	936 (38.60)	
Acute renal failure (N, %)					<0.001
No	1609 (66.46)	1755 (72.43)	2123 (87.69)	2362 (97.40)	
Yes	812 (33.54)	668 (27.57)	298 (12.31)	63 (2.60)	
Others					
Intubated (N, %)					<0.001
No	1855 (77.29)	1927 (79.99)	1911 (79.89)	1989 (83.19)	
Yes	545 (22.71)	482 (20.01)	481 (20.11)	402 (16.81)	
Mechanical ventilation use (N, %)					0.348
No	1675 (69.79)	1680 (69.74)	1669 (69.77)	1715 (71.73)	
Yes	725 (30.21)	729 (30.26)	723 (30.23)	676 (28.27)	
Dialysis (N, %)					<0.001
No	1933 (80.54)	2349 (97.51)	2382 (99.58)	2386 (99.79)	
Yes	467 (19.46)	60 (2.49)	10 (0.42)	5 (0.21)	
ICU 28-day mortality (N, %)					<0.001
No	2030 (83.85)	2140 (88.32)	2231 (92.15)	2321 (95.71)	
Yes	391 (16.15)	283 (11.68)	190 (7.85)	104 (4.29)	

Data are given as mean ± SD, median (interquartile range), or number (percentage).

P*P*: Kruskal Wallis test was used to compare non-normally distributed continuous variables between groups.

BMI, body mass index; bmp, beats per minute; MAP, mean arterial pressure; BNP, brain natriuretic peptide; PT, prothrombin time; INR, international normalized ratio; ESR, erythrocyte sedimentation rate; CRP, C-reactive protein; AST, aspartate aminotransferase; ALT, alanine aminotransferase; GCS, Glasgow Coma Scale; SOFA, Sequential Organ Failure Assessment; UTI, urinary tract infection; COPD, chronic obstructive pulmonary disease; CHF, congestive heart failure; AMI, acute myocardial infarction.

**Table 2:** Univariate analysis for 28-day mortality.

Covariate	Statistics	OR (95%CI) *p*-value
sACR	2.03 ± 1.41	0.65 (0.61, 0.70) <0.0001
Demographics		
Gender		
Male	4,784 (48.88%)	Ref.
Female	5,003 (51.12%)	1.02 (0.89, 1.16) 0.78
Age (years)	28.87 ± 8.86	0.99 (0.98, 1.00) 0.005
BMI (kg/m^2^)	24.74 ± 3.12	0.98 (0.94, 1.02) 0.335
Ethnicity		
Caucasian	7,549 (77.66%)	Ref.
African American	964 (9.92%)	1.00 (0.80, 1.26) 0.97
Hispanic	535 (5.50%)	1.01 (0.75, 1.35) 0.95
Asian	372 (3.83%)	0.92 (0.64, 1.33) 0.67
Native American	105 (1.08%)	1.19 (0.65, 2.17) 0.58
Other/Unknown	196 (2.02%)	1.34 (0.88, 2.06) 0.18
Vital signs		
Temperature (°C)	36.56 ± 1.28	0.83 (0.79, 0.87) <0.0001
Respiratory rate (bpm)	30.41 ± 14.35	1.02 (1.01, 1.02) <0.0001
Heart rate (/min)	112.56 ± 28.90	1.01 (1.01, 1.01) <0.0001
MAP (mmHg)	76.73 ± 43.10	1.00 (1.00, 1.00) 0.09
Laboratory data		
Lactate (mmol/l)	2.63 ± 2.44	1.26 (1.23, 1.29) <0.0001
Triglycerides (mg/dl)	175.32 ± 308.86	1.00 (1.00, 1.00) 0.66
Total cholesterol (mg/dl)	116.25 ± 53.62	0.99 (0.98, 1.00) 0.21
Albumin (g/dl)	2.50 ± 0.61	0.51 (0.46, 0.57) <0.0001
Serum creatinine (mg/dl)	2.09 ± 1.95	1.11 (1.08, 1.15) <0.0001
Sodium (mmol/l)	138.15 ± 6.35	1.01 (1.00, 1.02) 0.30
Calcium (mg/dl)	7.93 ± 0.88	0.85 (0.78, 0.92) <0.0001
Troponin I (ng/ml)	1.61 ± 6.36	1.01 (1.00, 1.03) 0.07
BNP (pg/ml)	3191.37 ± 9343.04	1.00 (1.00, 1.00) 0.09
CRP (mg/dl)	378.83 ± 791.60	1.00 (1.00, 1.00) 0.63
Blood urea nitrogen (mg/dl)	36.07 ± 26.41	1.01 (1.01, 1.01) <0.0001
PT - INR	1.94 ± 1.42	1.18 (1.13, 1.24) <0.0001
Platelets (x 10^9^/l)	196.27 ± 113.23	1.00 (1.00, 1.00) <0.0001
Hemoglobin (g/dl)	10.37 ± 2.15	0.97 (0.94, 1.00) 0.08
White blood cell count (×10^9^/l)	15.49 ± 12.19	1.01 (1.01, 1.01) <0.0001
ALT, U/L	115.52 ± 443.93	1.00 (1.00, 1.00) <0.0001
AST, U/L	186.44 ± 827.06	1.00 (1.00, 1.00) <0.0001
Severity of illness		
Acute physiology score III tertile		
4–46	2,710 (31.39%)	Ref.
47–66	2,994 (34.68%)	2.30 (1.77, 3.00) < 0.0001
67–177	2,930 (33.94%)	8.77 (6.90, 11.14) < 0.0001
SOFA score tertile	678 (61.41)	
0–2	2,554 (26.09%)	Ref.
3–5	3,599 (36.77%)	1.68 (1.32, 2.14) < 0.0001
6–21	3,635 (37.14%)	5.17 (4.16, 6.42) < 0.0001
Apache IV score tertile		
13–60	2,819 (32.65%)	Ref.
61–81	2,875 (33.30%)	2.70 (2.05, 3.55) < 0.0001
82–193	2,940 (34.05%)	9.99 (7.81, 12.80) < 0.0001
GCS score tertile		
3–11	2,812 (29.37%)	Ref.
12–14	2,591 (27.06%)	0.62 (0.53, 0.73) < 0.0001
15–15	4,173 (43.58%)	0.40 (0.34, 0.47) < 0.0001
Sepsis source of infection		
Pulmonary	3,692 (37.72%)	Ref.
Renal/UTI (including bladder)	2,206 (22.54%)	0.46 (0.37, 0.56) <0.0001
GI	1,380 (14.10%)	1.10 (0.91, 1.33) 0.32
Cutaneous/soft tissue	767 (7.84%)	0.54 (0.40, 0.72) <0.0001
Other	616 (6.29%)	0.74 (0.55, 0.99) 0.04
Gynecologic	29 (0.30%)	0.56 (0.13, 2.35) 0.43
Unknown	1,098 (11.22%)	1.00 (0.81, 1.24) 0.99
Comorbidities		
Hepatic failure		
No	9,435 (97.37%)	Ref.
Yes	255 (2.63%)	2.75 (2.04, 3.72) <0.0001
Metastatic cancer		
No	9,354 (96.53%)	Ref.
Yes	336 (3.47%)	1.81 (1.34, 2.43) <0.0001
Cirrhosis		
No	9,341 (96.40%)	Ref.
Yes	349 (3.60%)	2.73 (2.10, 3.54) <0.0001
Diabetes		
No	7,197 (74.27%)	Ref.
Yes	2,493 (25.73%)	0.78 (0.66, 0.92) 0.002
COPD		
No	9,037 (92.33%)	Ref.
Yes	751 (7.67%)	0.91 (0.70, 1.18) 0.48
CHF		
No	8,987 (91.82%)	Ref.
Yes	801 (8.18%)	1.26 (1.01, 1.58) 0.04
AMI		
No	9,462 (96.67%)	Ref.
Yes	326 (3.33%)	1.32 (0.94, 1.84) 0.11
Pneumonia		
No	6,592 (67.35%)	Ref.
Yes	3,196 (32.65%)	1.29 (1.12, 1.48) 0.0003
Others		
Intubated		
No	7,755 (80.03%)	Ref.
Yes	1935 (19.97%)	2.96 (2.57, 3.40) <0.0001
Mechanical ventilation use		
No	6,794 (70.11%)	Ref.
Yes	2,896 (29.89%)	2.36 (2.07, 2.71) <0.0001
Dialysis		
No	9,148 (94.41%)	Ref.
Yes	542 (5.59%)	1.13 (0.86, 1.49) 0.39

Data are given as mean ± SD, median (interquartile range), or percentage.

CI, confidence interval; OR, odds ratio.

### 3.2 Relationship between sACR and 28-day mortality in the different models

Table 2 presents the various factors associated with sACR and 28-day mortality using a univariate logistic model. The analysis indicated that sACR levels were negatively correlated with the risk of 28-day mortality (OR = 0.65, 95% CI: 0.61–0.70, *p* < 0.0001). Regarding disease severity scores, an increase in the Acute Physiology Score III and SOFA score was significantly associated with an elevated risk of mortality. It is noteworthy that the group with a SOFA score of 6–21 points exhibited a significantly elevated risk of mortality compared with the lowest-scoring group (0–2 points) (OR = 5.17, 95% CI: 4.16–6.42, *p* < 0.0001). Furthermore, an increase in the APACHE IV score was significantly correlated with a marked increase in the risk of death. The highest-scoring group (82–193 points) exhibited a markedly elevated risk of mortality compared with the lowest-scoring group (13–60 points). The OR was 9.99 (95% CI: 7.81–12.80, *p* < 0.0001).

**Table 3:** Relationship between sACR and 28-day mortality.

Exposure	Non-adjusted OR (95%CI) *p*-value	Model I OR (95%CI) *p*-value	Model II OR (95%CI) *p*-value
sACR	0.65 (0.61, 0.70) <0.0001	0.65 (0.61, 0.69) <0.0001	0.78 (0.71, 0.87) <0.0001
sACR (quartiles)			
Q1	Ref.	Ref.	Ref.
Q2	0.68 (0.58, 0.80) <0.0001	0.66 (0.56, 0.79) <0.0001	0.81 (0.64, 1.02) 0.07
Q3	0.43 (0.36, 0.52) <0.0001	0.42 (0.34, 0.50) <0.0001	0.63 (0.48, 0.84) 0.002
Q4	0.23 (0.19, 0.29) <0.0001	0.23 (0.18, 0.29) <0.0001	0.44 (0.31, 0.63) <0.0001
*p* for trend	<0.0001	<0.0001	<0.0001

Non-adjusted model adjust for: None. Model I adjusted for age, gender, BMI, ethnicity.

Model II adjusted for age, gender, BMI, ethnicity, temperature, respiratory rate, heart rate, MAP, blood urea nitrogen, calcium, AST, ALT, platelets, hemoglobin, white blood cell count, GCS score, SOFA score, COPD, CHF, AMI, diabetes, pneumonia, hepatic failure, metastatic cancer, intubated, mechanical ventilation use, dialysis.

Table 3 presents the findings of the multivariate logistic regression model for sACR and 28-day mortality. In Model I, after adjusting for age, sex, BMI, and race, the inverse correlation between sACR and 28-day mortality remained significant (OR = 0.65, 95% CI: 0.61–0.69, *p* < 0.0001). Furthermore, in Model II, when controlling for various potential confounding factors, including vital signs, laboratory data, disease severity scores, comorbidities, and treatment modalities, the correlation between sACR and 28-day mortality was somewhat attenuated but remained statistically significant (OR = 0.78, 95% CI: 0.71–0.87, *p* < 0.0001). Moreover, quartile analysis of sACR demonstrated that, in comparison with the lowest quartile (Q1), the risk of death was significantly reduced in the other three quartiles (Q2, Q3, and Q4) in the unadjusted model. After adjustment for Models I and II, the mortality risk increased in Q2, Q3, and Q4 but remained significantly lower than that in Q1. The trend test yielded a statistically significant result in all models (*p* < 0.0001), indicating a significant dose–response relationship between sACR and 28-day mortality Table 4.

**Table 4:** Threshold effect analysis of sACR and 28-day mortality using a two- piecewise regression model.

	Per 1-unit increase
Models	OR (95%CI) *p*-value
Model I	
One line effect	0.78 (0.71, 0.87) <0.0001
Model II	
Inflection point (K)	4.79
sACR < K	0.75 (0.67, 0.84) <0.0001
sACR > K	1.19 (0.72, 1.96) 0.49
*p* value for log likelihood ratio test	0.13

Adjusted: age, gender, BMI, ethnicity, temperature, respiratory rate, heart rate, MAP, blood urea nitrogen, calcium, AST, ALT, platelets, hemoglobin, white blood cell count, GCS score, SOFA score, COPD, CHF, AMI, diabetes, pneumonia, hepatic failure, metastatic cancer, intubated, mechanical ventilation use, dialysis.

### 3.3 Results of the stratified analysis

Considering the heterogeneity of the population, we conducted stratified regression analyses for all baseline indicators; the results are presented in Supplementary Table S1. The results indicated that the direction of the effect size was consistent across various subgroups, including sex, age, presence of intubation, mechanical ventilation, diabetes, and renal failure.

### 3.4 Analysis of the non-linear relationship between sACR and 28-day mortality

**Figure 2:**
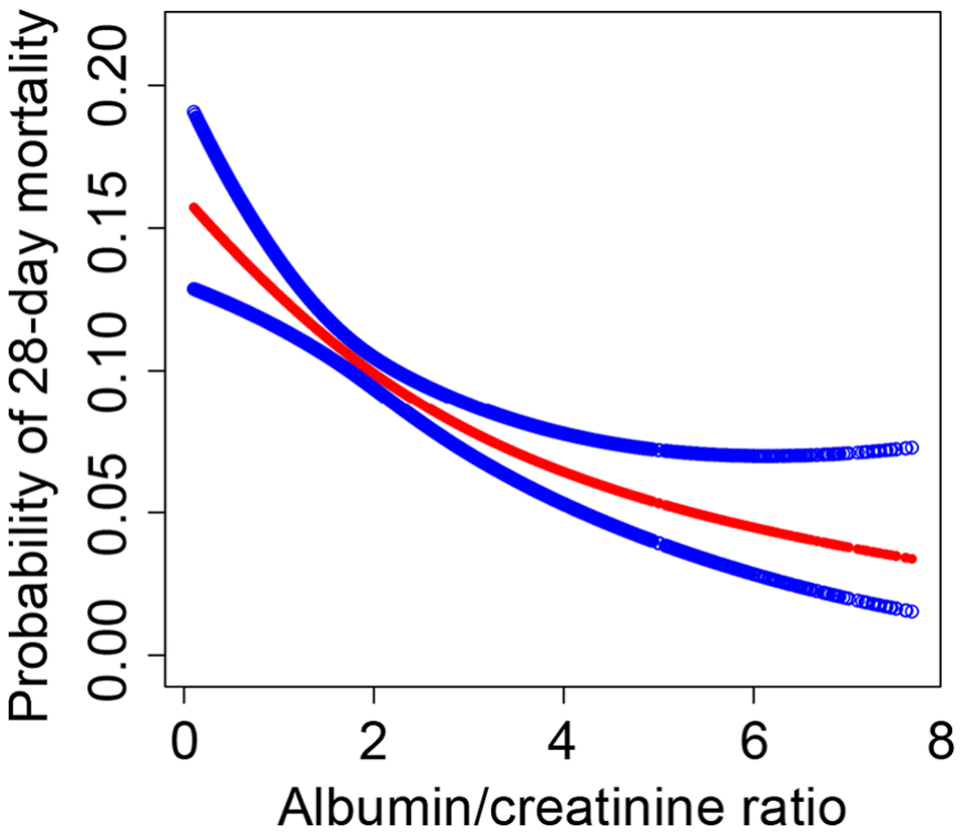
The association between the SACR and 28-day mortality in eICU patients with sepsis. A threshold, nonlinear association between SACR and 28-day mortality was found in a generalized additive model (GAM). Red line represents the smooth curve fit between variables. Blue lines represent the 95% of confidence interval from the fit. Adjusted for age, gender, BMI, ethnicity, temperature, respiratory rate, heart rate, MAP, blood urea nitrogen, calcium, AST, ALT, platelets, hemoglobin white blood cell count, GCS score, SOFA score, COPD, CHF, AMI, diabetes, pneumonia, hepatic failure, metastatic cancer, intubated, mechanical ventilation use, dialysis.

We investigated the potential non-linear correlation between sACR and 28-day mortality in the entire cohort using GAM and smooth curve fitting while accounting for variables such as age, sex, BMI, race, vital signs, and disease severity scores (Figure 2). As illustrated, there was a decreasing trend in 28-day mortality in the ICU with an increase in sACR, indicating a non-linear correlation.

The threshold effect of sACR on 28-day mortality was analyzed using a two-piecewise linear regression model. Model I demonstrates that for each unit increase in sACR, the OR for 28-day mortality was 0.78 (95% CI: 0.71–0.87), with a *p*-value <0.0001, indicating a significant association between sACR and the reduced risk of death. Model II demonstrated a threshold effect when the sACR index was less than 4.79, wherein a significant association was observed between sACR and a reduced risk of death (OR = 0.75, 95% CI: 0.67–0.84, *p* < 0.0001). Conversely, above this threshold, the observed association was not statistically significant (OR = 1.19, 95% CI: 0.72–1.96, *p* = 0.49). Several covariates, including age, sex, and BMI, were adjusted for in the analysis. The results demonstrated a non-linear relationship between sACR and 28-day mortality rate, with a particularly pronounced association below a specific threshold.

## 4 Discussion

This retrospective cohort study identified a non-linear correlation between sACR and 28-day ICU mortality, with a threshold effect, within the eICU-CRD database comprising 208 different ICUs across the United States between 2014 and 2015. Thus, this study’s findings indicate that sACR is an independent predictor of 28-day mortality. To our best knowledge, this is the first study to report an association between sACR and 28-day mortality in ICU patients with sepsis.

sACR has also been identified as a novel biomarker (11). Prior research has demonstrated a strong correlation between sACR and unfavorable outcomes in cardiovascular diseases, with an increasing body of evidence emerging for its role in other diseases. For example, Li et al. reported that a lower sACR was significantly correlated with a higher risk of death in patients with heart failure (17). This finding remained significant in the multivariate analysis, indicating that sACR was an independent risk factor for mortality. Türkyılmaz et al. found that a decrease in sACR was associated with the deterioration of short-term clinical outcomes, including 30-day mortality and other cardiac events, in patients with ST-segment elevation myocardial infarction (18). Kong et al. further elucidated the clinical significance of sACR, demonstrating that in patients with ST-elevation myocardial infarction undergoing percutaneous coronary intervention, a low sACR was significantly associated with an increased risk of pulmonary infection and major adverse cardiovascular events during hospitalization. In addition, they established that sACR was an independent predictor of long-term all-cause mortality (19). These findings highlight the potential role of sACR in assessing the risk of complications after cardiac surgery. Lastly, Nseir et al. demonstrated that sACR values were predictive factors for 30-day all-cause mortality in patients with *Clostridium difficile*-associated diarrhea, indicating that sACR may also have potential applications in the prognostic assessment of non-cardiac diseases (20). On the basis of these findings, we postulated that sACR might also have important clinical value in the assessment of other diseases. To our best knowledge, no previous study has explored the sACR values that are associated with the lowest mortality risk in patients with sepsis. Our findings lend further support to the existing view as set forth in the literature.

Our investigation revealed a negative correlation between sACR and 28-day mortality rate in ICU patients with sepsis. In addition, we conducted stratified regression analyses on the basis of various baseline indicators to assess the effect of population heterogeneity on this relationship and found that the negative correlation remained stable. However, the relationship was not linear. The principal finding was that sACR exhibited a more pronounced correlation with 28-day mortality below the threshold of 4.79; however, the correlation was no longer statistically significant at levels exceeding this threshold. The insights derived from this study contribute a novel dimension to the existing literature, enhancing our understanding of the dose–response relationship between sACR and survival rates of patients with sepsis. They can provide clinicians with more precise guidance when assessing patient risks.

The precise mechanism underlying this study’s findings remains unclear. In light of previous clinical research, we postulated that the impact of sACR levels on the 28-day ICU mortality rate in patients with sepsis may be mediated through the following pathways. First, low sACR may indicate malnutrition, which can affect immune function and lead to multiorgan dysfunction, increasing the risk of death (21). Second, low sACR may indicate an inflammatory response that can hinder albumin synthesis and accelerate its metabolism, resulting in a decrease in plasma colloid osmotic pressure and insufficient blood circulation, which affects organ perfusion and function (22). Third, serum albumin plays a role in the protection of vascular endothelial cells through its antioxidant effects, which are exerted through the capture of reactive oxygen and nitrogen species by its thiol moiety as well as through the binding of metal ions and fatty acids. Consequently, oxidative stress-related vascular injuries may be exacerbated in patients with serum albumin deficiency (23, 24). Fourth, serum albumin deficiency may result in a reduction in the anticoagulant capacity of blood, thereby increasing the likelihood of clot formation. This hypercoagulable state may be associated with an elevated risk of mortality, particularly in critically ill patients (25, 26). Fifth, increased creatinine levels are markers of kidney injury, and the pathophysiological mechanisms of sepsis-associated acute kidney injury, including microvascular dysfunction, inflammation, and metabolic reprogramming, may lead to decreased kidney function and increased mortality (27). Further research is required to fully elucidate the pathophysiological importance of sACR.

The present study also has the following limitations. (1) As a retrospective cohort study, it was only able to establish associations rather than causality. Given the observational nature of our study, other unmeasured confounding factors may have influenced the results. Therefore, large-scale prospective studies should be conducted in the future to verify the findings presented here. (2) Serum albumin and creatinine levels were determined within the initial 24-h period following ICU admission. It is possible that these initial values do not fully reflect the changes in renal function or albumin levels that may occur during the ICU stay. (3) It is challenging to determine whether sepsis is the primary cause of ICU admission. However, it should be noted that ICU admissions are typically driven by critical conditions associated with multiple diseases, including sepsis. (4) The present study used data from the U.S. eICU database, which may limit the generalizability of its findings to other populations.

## 5 Conclusion

This study established that low sACR values are independently associated with an increased 28-day mortality rate in ICU patients with sepsis, exhibiting a non-linear dose–response relationship and a threshold effect. These findings may serve as early warning indicators in high-risk populations.

## Data Availability

The datasets presented in this study can be found in online repositories. The names of the repository/repositories and accession number(s) can be found below: https://physionet.org/content/eicu-crd/2.0/.

## Footnotes

^1^ http://www.empowerstats.com
