# Supplementary material for: Association between the serum albumin–creatinine ratio and 28-day intensive care unit mortality among patients with sepsis: a multicenter retrospective cohort study
Source: Front Med (Lausanne). 2024 Nov 5;11:1484370. doi: 10.3389/fmed.2024.1484370 (PMC11573561; doi:10.3389/fmed.2024.1484370)
Supplement: Supplementary file 1 [file Table_1.docx]

**Table S1** Stratified analyese of the association 28-day mortality and sACR

| Subgroup | Participants | sACR, OR (95% Cl) | P-value |
| --- | --- | --- | --- |
| Age |  |  |  |
| ≤ 67.00 | 4679 | 0.68 (0.62, 0.74) | <0.001 |
| > 67.00 | 5011 | 0.63 (0.57, 0.69) | <0.001 |
| Gender |  |  |  |
| Male | 4715 | 0.71 (0.65, 0.77) | <0.001 |
| Female | 4974 | 0.58 (0.53, 0.64) | <0.001 |
| Ethnicity recoded |  |  |  |
| caucasian | 7485 | 0.66 (0.62, 0.71) | <0.001 |
| non-caucasian | 2138 | 0.61 (0.53, 0.70) | <0.001 |
| BMI |  |  |  |
| ≤ 27.20 | 4724 | 0.69 (0.64, 0.75) | <0.001 |
| > 27.20 | 4772 | 0.59 (0.53, 0.65) | <0.001 |
| Temperature |  |  |  |
| ≤ 36.50 | 4321 | 0.67 (0.61, 0.73) | <0.001 |
| > 36.50 | 4855 | 0.67 (0.61, 0.74) | <0.001 |
| Respiratory rate |  |  |  |
| ≤ 32.00 | 4600 | 0.57 (0.51, 0.64) | <0.001 |
| > 32.00 | 4964 | 0.69 (0.64, 0.75) | <0.001 |
| Heart rate |  |  |  |
| ≤ 114.00 | 4633 | 0.65 (0.58, 0.72) | <0.001 |
| > 114.00 | 4952 | 0.65 (0.60, 0.70) | <0.001 |
| MAP |  |  |  |
| ≤ 56.00 | 4695 | 0.67 (0.61, 0.73) | <0.001 |
| > 56.00 | 4883 | 0.68 (0.62, 0.74) | <0.001 |
| Lactate |  |  |  |
| ≤ 1.90 | 3173 | 0.72 (0.64, 0.82) | <0.001 |
| > 1.90 | 3265 | 0.63 (0.57, 0.70) | <0.001 |
| Triglycerides |  |  |  |
| ≤ 121.00 | 378 | 0.63 (0.46, 0.87) | 0.005 |
| > 121.00 | 379 | 0.69 (0.48, 0.98) | 0.036 |
| Albumin |  |  |  |
| ≤ 2.50 | 4689 | 0.65 (0.59, 0.72) | <0.001 |
| > 2.50 | 5001 | 0.71 (0.65, 0.78) | <0.001 |
| Serum creatinine |  |  |  |
| ≤ 1.41 | 4727 | 0.68 (0.61, 0.77) | <0.001 |
| > 1.41 | 4963 | 0.63 (0.53, 0.75) | <0.001 |
| Sodium |  |  |  |
| ≤ 138.00 | 4410 | 0.65 (0.60, 0.72) | <0.001 |
| > 138.00 | 5278 | 0.65 (0.59, 0.71) | <0.001 |
| Calcium |  |  |  |
| ≤ 7.90 | 4470 | 0.64 (0.58, 0.71) | <0.001 |
| > 7.90 | 5107 | 0.67 (0.62, 0.74) | <0.001 |
| Troponin I |  |  |  |
| ≤ 0.15 | 1494 | 0.73 (0.64, 0.84) | <0.001 |
| > 0.15 | 1586 | 0.83 (0.72, 0.95) | 0.007 |
| BNP |  |  |  |
| ≤ 639.00 | 419 | 0.50 (0.34, 0.73) | <0.001 |
| > 639.00 | 420 | 0.73 (0.56, 0.97) | 0.031 |
| Blood urea nitrogen |  |  |  |
| ≤ 29.00 | 4699 | 0.69 (0.62, 0.76) | <0.001 |
| > 29.00 | 4982 | 0.77 (0.69, 0.86) | <0.001 |
| PT - INR |  |  |  |
| ≤ 1.50 | 2281 | 0.80 (0.70, 0.90) | <0.001 |
| > 1.50 | 2379 | 0.64 (0.57, 0.73) | <0.001 |
| Platelets |  |  |  |
| ≤ 178.00 | 4709 | 0.59 (0.53, 0.65) | <0.001 |
| > 178.00 | 4701 | 0.73 (0.67, 0.80) | <0.001 |
| Hemoglobin |  |  |  |
| ≤ 10.20 | 4586 | 0.78 (0.72, 0.85) | <0.001 |
| > 10.20 | 4881 | 0.55 (0.50, 0.61) | <0.001 |
| White blood cell |  |  |  |
| ≤ 13.30 | 4729 | 0.64 (0.59, 0.71) | <0.001 |
| > 13.30 | 4756 | 0.68 (0.62, 0.74) | <0.001 |
| ESR |  |  |  |
| ≤ 48.00 | 172 | 0.52 (0.31, 0.86) | 0.010 |
| > 48.00 | 178 | 0.67 (0.38, 1.16) | 0.153 |
| CRP |  |  |  |
| ≤ 20.75 | 150 | 0.84 (0.53, 1.34) | 0.464 |
| > 20.75 | 156 | 0.90 (0.54, 1.50) | 0.692 |
| ALT |  |  |  |
| ≤ 27.00 | 4256 | 0.71 (0.64, 0.78) | <0.001 |
| > 27.00 | 4622 | 0.62 (0.57, 0.68) | <0.001 |
| AST |  |  |  |
| ≤ 36.00 | 4393 | 0.77 (0.70, 0.86) | <0.001 |
| > 36.00 | 4565 | 0.62 (0.57, 0.68) | <0.001 |
| Acute Physiology Score III |  |  |  |
| ≤ 57.00 | 4264 | 0.82 (0.73, 0.93) | 0.002 |
| > 57.00 | 4286 | 0.77 (0.71, 0.83) | <0.001 |
| SOFA score |  |  |  |
| ≤ 4.00 | 3645 | 0.72 (0.62, 0.82) | <0.001 |
| > 4.00 | 6045 | 0.75 (0.70, 0.81) | <0.001 |
| Apache IV score |  |  |  |
| ≤ 71.00 | 4144 | 0.88 (0.77, 0.99) | 0.034 |
| > 71.00 | 4406 | 0.76 (0.70, 0.83) | <0.001 |
| GCS score |  |  |  |
| ≤ 14.00 | 3900 | 0.67 (0.62, 0.73) | <0.001 |
| > 14.00 | 5579 | 0.64 (0.58, 0.71) | <0.001 |
| Sepsis source of infection |  |  |  |
| renal | 7509 | 0.65 (0.61, 0.70) | <0.001 |
| non-renal | 2181 | 0.59 (0.48, 0.72) | <0.001 |
| Acute renal failure |  |  |  |
| No | 7849 | 0.67 (0.62, 0.72) | <0.001 |
| Yes | 1841 | 0.62 (0.50, 0.77) | <0.001 |
| Hepatic failure |  |  |  |
| No | 9338 | 0.65 (0.61, 0.70) | <0.001 |
| Yes | 254 | 0.78 (0.59, 1.03) | 0.082 |
| Metastatic cancer |  |  |  |
| No | 9256 | 0.66 (0.61, 0.70) | <0.001 |
| Yes | 336 | 0.60 (0.45, 0.81) | <0.001 |
| Cirrhosis |  |  |  |
| No | 9245 | 0.65 (0.61, 0.70) | <0.001 |
| Yes | 347 | 0.77 (0.60, 0.98) | 0.033 |
| Diabetes |  |  |  |
| No | 7115 | 0.66 (0.62, 0.71) | <0.001 |
| Yes | 2477 | 0.56 (0.47, 0.66) | <0.001 |
| COPD |  |  |  |
| No | 8945 | 0.64 (0.60, 0.68) | <0.001 |
| Yes | 745 | 0.81 (0.66, 0.99) | 0.035 |
| CHF |  |  |  |
| No | 8891 | 0.64 (0.60, 0.69) | <0.001 |
| Yes | 799 | 0.79 (0.64, 0.99) | 0.036 |
| AMI |  |  |  |
| No | 9367 | 0.66 (0.62, 0.70) | <0.001 |
| Yes | 323 | 0.50 (0.33, 0.76) | 0.001 |
| Pneumonia |  |  |  |
| No | 6540 | 0.59 (0.54, 0.65) | <0.001 |
| Yes | 3150 | 0.71 (0.64, 0.78) | <0.001 |
| Intubated |  |  |  |
| No | 7682 | 0.64 (0.59, 0.69) | <0.001 |
| Yes | 1910 | 0.71 (0.64, 0.79) | <0.001 |
| Mechanical ventilation use |  |  |  |
| No | 6739 | 0.65 (0.60, 0.71) | <0.001 |
| Yes | 2853 | 0.65 (0.59, 0.72) | <0.001 |
| Dialysis |  |  |  |
| No | 9050 | 0.63 (0.59, 0.68) | <0.001 |
| Yes | 542 | 1.00 (0.58, 1.73) | 0.997 |

Non-adjusted model adjust for: None.

Abbreviations: OR odd ratio, CI confdence interval
